# 非小细胞肺癌*EGFR*突变的分子影像学在体检测

**DOI:** 10.3779/j.issn.1009-3419.2017.06.08

**Published:** 2017-06-20

**Authors:** 丹静 罗, 进安 马, 锦明 张, 颜忠 赵

**Affiliations:** 1 410011 长沙，中南大学湘雅二医院肿瘤中心 Department of Oncology, The Second Xiangya Hospital, Center South University, Changsha 410011, China; 2 100853 北京，中国人民解放军总医院核医学科 Department of Nuclear Medicine, The PLA General Hospital, Beijing 100853, China; 3 410013 长沙，中南大学湘雅三医院医学实验中心 The Medical Experimental Center, the Third Xiangya Hospital, Central South University, Changsha 410013, China

**Keywords:** 肺肿瘤, 表皮生长因子受体, 正电子发射计算机断层显像, 分子探针, Lung neoplasms, EGFR, PET-CT, Molecular probe

## Abstract

靶向药物表皮生长因子受体酪氨酸激酶抑制剂（epidermal growth factor receptor tyrosine kinase inhibitor, EGFR-TKI）改变了非小细胞肺癌的治疗格局，研究表明只有*EGFR*敏感突变人群能从中获益。*EGFR*突变检测的主流方法是针对EGFR的DNA序列进行分析，标本可以是手术或穿刺获取的肺癌组织、胸水肿瘤细胞、循环肿瘤细胞、外周血游离DNA，其最大的缺点是无法分析*EGFR*突变的异质性。针对EGFR在蛋白质水平进行突变检测分析的技术尚不成熟，但随着分子影像学的发展，基于正电子发射型计算机断层显像（positron emission computed tomography, PET）-计算机断层扫描（computed tomography, CT）的靶向EGFR分子探针的研发，使得在体检测肺癌组织的*EGFR*突变状态成为了可能，而且可以检测*EGFR*突变的异质性。本文综述了目前靶向*EGFR*突变的分子探针的研究结果及进展。

## Abstract (en)

An ever increasing number of drugs directed as epidermal growth factor receptor tyrosine kinase inhibitor (EGFR-TKI) bring a new revolution for non-small cell lung cancer (NSCLC) therapy, and many large scales of studies show that only people with *EGFR*-sensitive mutation can benefit from these drugs. The main method of *EGFR* mutation detection is to analyze the DNA sequence of EGFR, which can be the lung cancer tissue, pleural fluid tumor cells, circulating tumor cells and peripheral blood free DNA obtained by surgery or puncture, the biggest drawback is that the heterogeneity of *EGFR* mutation cannot be analyzed. However, with the development of molecular imaging, the development of EGFR-targeted molecular probes based on positron emission computed tomography-computed tomography (PET-CT) has made it possible to reveal the *EGFR* mutations in lung cancer tissues *in vivo*, and can detect the heterogeneity of *EGFR* mutations. This article reviews all the results and progress of molecular probes targeting *EGFR* mutations.

晚期非小细胞肺癌（non-small cell lung cancer, NSCLC）的治疗真正进入了分子分型时代，患者自身疾病的分子特征和治疗密切相关，并需要以此为依据来选择相应的分子靶向药物。基于驱动基因的靶向药物不断出现，尤其是表皮生长因子受体酪氨酸激酶抑制剂（epidermal growth factor receptor tyrosine kinase inhibitor, EGFR-TKI），*EGFR*突变NSCLC患者有了越来越多的选择。近十年来，EGFR-TKI的研发和广泛应用，明显提高了突变患者的总生存期（overall survival, OS）。但所有数据都表明只有*EGFR*敏感突变人群能从中获益，因此*EGFR*的突变状态成为临床使用此类药物的重要前提，其突变检测结果显得尤为重要。现有检测的主流方法是针对EGFR的DNA序列进行分析，标本可以是手术或穿刺获取的肺癌组织、胸水肿瘤细胞、循环肿瘤细胞、外周血游离DNA，其最大的缺点是无法分析*EGFR*突变的异质性，从而一定程度上影响了*EGFR*敏感突变肺癌患者使用EGFR-TKI的有效率^[1]^。但随着分子影像学的发展，基于正电子发射型计算机断层显像（positron emission computed tomography, PET）-计算机断层扫描（computed tomography, CT）的靶向EGFR分子探针的研发，使得在体检测肺癌组织的*EGFR*突变状态成为了可能，同时可以检测*EGFR*突变的异质性。本文综述了目前靶向*EGFR*突变的分子探针的研究结果及进展。

## 1 *EGFR*基因突变

EGFR属于酪氨酸激酶受体家族，由细胞外配体结合域、疏水跨膜结构域和细胞内酪氨酸激酶结构域3个部分组成，通过与天然配体结合形成二聚体，使胞内酪氨酸激酶磷酸化，启动下游信号转导通路来调节细胞凋亡、增殖、分化等。*EGFR*基因突变主要发生在编码细胞内酪氨酸激酶结构域的18-21外显子，最常见的突变为19外显子的缺失性突变和21外显子的L858R点突变。这些突变基因编码的*EGFR*突变蛋白可不依赖于配体的结合，直接导致胞内酪氨酸激酶自动磷酸化，启动下游信号转导通路^[2]^。

## 2 EGFR相关的靶向治疗

针对EGFR的分子靶向药物，按其性质主要分为两大类：一类是单克隆抗体，直接阻断EGFR与配体结合，从而阻断下游信号转导通路，如西妥昔单抗、帕尼单抗、尼妥珠单抗，但临床研究^[3, 4]^表明EGFR单克隆抗体在NSCLC的靶向治疗上疗效有限，其治疗效果和EGFR突变状态及表达水平有关。另一类是小分子酪氨酸激酶抑制剂EGFR-TKI，在细胞内酪氨酸激酶结构域上与ATP竞争性结合，抑制下游信号转导，如吉非替尼、厄洛替尼、阿法替尼、奥希替尼。对于*EGFR*敏感突变阳性的晚期肺癌患者，IPASS、NEJ002、WJ0G3405、OPTIMAL、LUX-Lung3/LUX-Lung6、AURA3等^[5-10]^Ⅲ期临床研究先后证实，相比传统化疗，EGFR-TKI明显的延长了该类患者的生存期，也改善了患者的生活质量，具有良好的安全性。与此同时，IPASS、CTONG0806等^[5, 10-12]^临床研究显示*EGFR*野生型患者并不能从EGFR-TKI使用中获益，未降低该类患者的疾病进展或死亡风险，故不推荐使用。

由此可见，*EGFR*基因突变是一线EGFR-TKI治疗受益的先决条件，EGFR-TKI的使用需以*EGFR*基因突变检测为基础已达成全球共识^[13-15]^。

## 3 *EGFR*基因突变传统的检测现状

目前*EGFR*突变的检测方法以组织检查为金标准，现在临床最常用的为基于实时PCR的检测方法，优点是准确可靠、特异性高、检测周期相对缩短，缺点是检测结果受组织量多少和肿瘤时空异质性的影响。细胞学检测及外周血*EGFR*突变检测作为组织标本突变检测的补充，也已经有了良好的发展。而细胞学检测需要检测方法灵敏度较高、实验室条件严格和足够细胞标本，虽然特异性高，但检出率低。外周血*EGFR*突变的检测与组织*EGFR*突变检测有较高的符合率，特异性高，但其敏感性不够。二代测序虽然可以提高外周血检测的灵敏度，但存在检测周期长、费用昂贵等缺点，暂时无法广泛应用于临床。

## 4 EGFR相关分子探针示踪的PET-CT在体检测

无论是基于肺癌组织、细胞学、还是基于外周血的游离肿瘤DNA片段的*EGFR*突变检测，均存在或多或少的局限性，尤其是时空异质性的问题无法解决。故现在急需一种能够实时、在体检测EGFR表达水平或突变状态的方法，以指导EGFR-TKI临床治疗。分子显像技术可以在活体状态下，通过将参与基因、蛋白的表达、物质代谢等过程中重要化合物及分子进行放射性标记或荧光标记，利用PET-CT、PET-MR或者SPECT等影像学技术，直接将复杂的生物过程变成实时、动态的图像，实现组织、细胞和分子水平的定性和定量研究。而EGFR作为肺癌的特异性靶点，如若标记相应的靶向药物，与蛋白受体上胞外结构域或胞内特定结构域相结合，再利用PET显像仪器探测放射性物质形成相应的图像，从摄取放射性物质的高低就可以直观的反应出EGFR的表达水平或突变状态。目前已有多位学者进行了这类分子探针的研究，一种是标记EGFR的单克隆抗体如cetuximab、Panitumumab，另一种是标记小分子EGFR-TKI如吉非替尼、厄洛替尼、阿法替尼，未上市的PD153035等。

### 4.1 单克隆抗体类分子探针

单克隆抗体直接靶向EGFR细胞外结构域，阻断EGFR与配体结合，从而阻断下游信号转导通路。单克隆抗体均为大分子，渗透进组织较慢，故标记时需要半衰期相对较长的核素，如^64^Cu、^111^In、^89^Zr。

#### 4.1.1 西妥昔单抗（cetuximab）

西妥昔单抗是第一个经食品药品监督管理局（Food and Drug Administration, FDA）批准上市的EGFR单克隆抗体。Perk等^[16]^首次利用^89^Zr构建了靶向EGFR胞外配体结合域的分子探针^89^Zr-cetuximab，并在细胞A431移植瘤模型中发现EGFR高表达的肿瘤组织摄取明显高于非肿瘤组织。随后多位学者^[17, 18]^用不同核素标记cetuximab，并用合成的分子探针在相应的荷瘤鼠上进行了显像研究，均表明EGFR高表达水平区域的放射性浓聚高于周围组织。但Niu等^[19]^合成了^64^Cu-DOTA-cetuximab进行显像后，未发现上述规律，反而原本摄取应该低的UM-SCC-22B（EGFR表达水平低）细胞有较高的摄取，考虑为血管密度和血管通透性的影响，有待进一步证实。

#### 4.1.2 帕尼单抗（Panitumumab）

Nayak等^[20, 21]^则标记合成了^86^Y-CHX-A’’-DTPA-Panitumumab和^89^Zr-Panitumumab，进行不同EGFR表达水平移植瘤模型研究，得到的结果也是EFGR表达高的区域存在放射性浓聚，摄取明显高于EGFR表达低的区域。

上述两种单克隆抗体类EGFR分子探针的相关研究充分说明其在体检测EGFR蛋白表达水平的可行性，但存在很多问题，首先该类探针作为大分子蛋白在核素标记过程中易发生变性或降解，还可能与正常组织发生交叉免疫反应，所以导致该类研究局限于细胞和动物研究，限制了其在临床方面的应用。再者就是*EGFR*基因突变主要发生在胞内酪氨酸激酶结构域，所以此类探针不能检测*EGFR*是否存在突变。

### 4.2 EGFR-TKI类分子探针

EGFR-TKI进行放射性标记后，可以与突变蛋白的胞内酪氨酸酶结构域特异性结合，而与未发生突变的蛋白结合相对较少，从摄取的高低就可以直观地反映出EGFR表达水平和突变状态，理论上明显优于单克隆抗体类分子探针。此类小分子物质探针渗入细胞快，所用标记核素半衰期较短，如^11^C、^18^F。

#### 4.2.1 PD153035

PD153035是一种未上市的强效第一代EGFR-TKI，也是最先被研究的小分子酪氨酸酶抑制剂，对EGFR酪氨酸激酶的半抑制浓度（half maximal inhibitory concentration, IC_50_）为29 pmol/L，可在其6位或7位C上标记11C后生成11C-PD153035，不改变其化学结构和生化性质。

Dai^[22]^研究了^11^C-PD153035在三种肺癌细胞HCC827、A549、PC9中的摄取，发现有敏感突变的细胞株HCC827摄取高于其他细胞株，此体外细胞研究证实了^11^C-PD153035检测EGFR蛋白表达水平及*EGFR*突变的可能性；而Wang等^[23]^通过3种不同EGFR蛋白表达水平的肺癌移植瘤模型，从动物层面进一步证实了^11^C-PD153035的摄取与肿瘤细胞的EGFR蛋白表达水平成正相关；而Liu等^[24]^在正常人体内进行了相关研究，发现^11^C-PD153035在体内以肝和肾代谢为主，放射性核素的辐射剂量处在安全范围，这显然为临床推广应用奠定了生理基础；国内学者Meng等^[25]^进一步进行了21例NSCLC患者的临床研究，认为其摄取状态和EGFR表达水平成正相关，并且EGFR表达水平和患者的无进展生存期（progression-free survival, PFS）、总生存期（overall survival, OS）密切相关，从而表明^11^C-PD153035分子显像可以作为临床疗效的预测手段，指导临床靶向药物的使用。

^11^C-PD153035细胞、动物、临床三个方面的研究表明了此类小分子EGFR-TKI探针成像和临床推广的可能性，但研究多侧重于检测EGFR蛋白的表达水平，同时有研究发现PD153035对某些细胞系如A431有一定的毒性。因此，研究者们开始考虑标记其类似物如吉非替尼、厄洛替尼、阿法替尼等进行研究。

#### 4.2.2 吉非替尼

吉非替尼（gefitinib）是ATP竞争性的EGFR酪氨酸激酶可逆型抑制剂，阻断细胞信号的传递，抑制细胞异常增生和转移，2003年FDA批准用于临床。从结构上看，吉非替尼既可以做11C标记，也可以用18F标记而不改变其生化性质。

Zhang等^[26]^和张锦明等^[27]^用^11^C标记合成了^11^C-gefitinib，从细胞及动物两个层面进行了相关研究，前者研究发现高EGFR表达水平的A431细胞株的放射性摄取明显高于低EGFR表达水平的Jurkat细胞，并在荷NFSa肿瘤的裸鼠模型上显示肿瘤区有^11^C-gefitinib特异性地浓聚，后者则进一步证实^11^C-gefitinib为特异性摄取，通过建立A549荷瘤裸鼠模型，发现有^11^C-gefitinib放射性浓聚的肿瘤区经gefitinib标准品阻断后，放射性摄取明显下降。

而Su等^[28]^用^18^F标记合成了^18^F-gefitinib，研究却得到了不同的结果，合成的^18^F-gefitinib在4种不同肿瘤细胞（低表达EGFR U87，高表达EGFR U87-EGFR，含L858R突变H3255，含L858R突变合并T790M突变H1975）中均未发现肿瘤区有放射性物质的特异性浓聚。考虑可能是由于其亲脂性高，与靶点可逆结合，从而肿瘤区积累过慢或排除过快所致。

^11^C-gefitinib显像研究发现其均能与突变型*EGFR*和野生型EGFR结合，故可以检测EGFR蛋白表达水平，但在检测*EGFR*突变方面无明显优势。而^18^F-gefitinib不能显像，与^11^C-gefitinib的研究结果不一致，具体原因尚不清楚。

#### 4.2.3 厄洛替尼

厄洛替尼（erlotinib）也是一种经FDA认证的喹唑啉类衍生物，与EGFR的活性构象可逆结合，能高效且特异性的抑制EGFR酪氨酸激酶。它对突变的EGFR酪氨酸激酶的IC_50_达到了2 nmol/L。从结构上看，^11^C标记6-氧-去甲基erlotinib最佳。Memon等^[29]^首次利用^11^C合成了^11^C-erlotinib，建立HCC827细胞（外显子19缺失突变）和A549、NCI358细胞（*EGFR*野生型）三种荷瘤裸鼠模型，PET扫描和生物分布实验结果显示，^11^C-erlotinib在HCC827肿瘤模型中摄取最高，持续时间最长，在肿瘤组织区浓聚，而周围组织无或轻度摄取，而A549和NCI358裸鼠模型则无放射性浓聚，提示^11^C-erlotinib可用于检测*EGFR*突变状态，筛选*EGFR*突变人群。该结果被Abourbeh^[30]^的研究再一次证实。而Petrulli^[31]^的研究则进一步支持^11^C-erlotinib能和突变的*EGFR*结合显像，该实验通过对PC9（EGFR野生型）、HCC827（19外显子缺失）、U87（胶质瘤细胞）、U87-EGFR（表达EGFR的胶质瘤细胞），SW620（肠癌细胞）5种荷瘤裸鼠模型进行PET显像，并进行未标记的厄洛替尼阻断竞争实验，发现原本高摄取的肿瘤区在被阻断后并不显影。

在上述细胞及动物实验的基础上，研究者们将眼光直接投向了临床应用。尤其是Weber等^[32]^直接对1例NSCLC脑转移患者进行^11^C-erlotinib显像研究，发现脑转移灶有明显的放射性浓聚，和周围脑实质对比清晰，不仅证实了^11^C-erlotinib能对原发肿瘤显像，还可以在脑转移灶中显像。Memon^[33]^对13例NSCLC患者厄洛替尼治疗前后行^11^C-erlotinib PET/CT和^18^F-FDG PET/CT扫描，证实^11^C-erlotinib PET/CT可以检测出^18^F-FDG PET/CT未检出的淋巴结转移灶，更加巩固了^11^C-erlotinib显像的优势。

为了进一步证实^11^C-erlotinib与*EGFR*突变蛋白是特异性结合而显像，Bahce等^[34, 35]^进行了两项研究，第一项通过对已知*EGFR*突变状态的10例NSCLC患者（5例19外显子缺失突变，5例没有突变）中进行^11^C-erlotinib的显像研究，结果表明*EGFR*突变患者肿瘤^11^C-erlotinib的分布容积明显高于非突变组，证实了^11^C-erlotinib与*EGFR*突变蛋白的结合；后一项实验则是对已知*EGFR*突变状态的10例NSCLC患者行厄洛替尼治疗前后的^11^C-erlotinib PET/CT扫描研究，结果显示服用厄洛替尼有效的患者对^11^C-erlotinib的摄取明显减少，进一步说明*EGFR*突变与^11^C-erlotinib结合的特异性，可以通过其放射性摄取高低预测厄洛替尼的疗效，以及通过其放射性摄取的变化监测临床靶向药物的疗效。

^11^C-erlotinib的研究不管是从细胞、动物还是临床方面均证实其对*EGFR*突变蛋白显像有一定的特异性，不仅可以筛选突变人群，指导靶向治疗，还能作为疗效监测的一种手段。上述三种靶向药物均为EGFR酪氨酸激酶的可逆抑制剂，虽然在检测EGFR蛋白表达水平或突变上有一定的作用，但仍存在许多的不足，如与EGFR受体为可逆性结合，加上机体代谢的影响，导致放射性核素的注射量无法确定，同时不能对存在原发性T790M突变的NSCLC进行显像。

#### 4.2.4 阿法替尼

阿法替尼（afatinib）是不可逆型EGFR酪氨酸激酶抑制剂，也是竞争性结合EGFR酸氨酸激酶胞内区的ATP结合位点，与可逆型抑制剂不同的是，其在结构上融合了可以与EGFR酪氨酸激酶结合口袋开口处附近所特有的氨基酸残基（Cys797）发生烷基化作用或共价键结合的基团，使其共价结合EGFR酪氨酸激酶而不离去，从而对EGFR酪氨酸激酶产生不可逆地抑制，而且对EGFR的T790M突变也具有良好的抑制能力。从结构上看，其不仅能采用^11^C进行标记，还可采用^18^F进行标记。但其是泛EGFR家族的抑制剂，还可作用于HER-2、HER-4，这个多靶点特性理论上可能会限制其作为分子探针的应用。

Slobbe等^[36]^直接合成了^18^F-afatinib和^11^C-erlotinib进行对比研究，共建立了A549（EGFR野生型）、H1975（L858R/T790M突变）、HCC827（19外显子缺失突变）3种荷瘤裸鼠模型，PET成像结果显示，^11^C-erlotinib显像剂在HCC827细胞荷瘤鼠中显示出肿瘤区放射性浓聚高于周围组织，而在无突变的A549、L858R突变合并T790M突变的移植瘤中均不显影，而^18^F-afatinib在3种细胞株的移植瘤中均显示滞留良好，这与阿法替尼对*EGFR*突变型和野生型NSCLC细胞均起作用相符（对21外显子L858R突变的EGFR IC_50_为0.4 nmol/L，L858R/T790M突变的IC_50_为10 nmol/L，野生型的IC_50_仅为0.5 nmol/L）。由此可见第二代TKI阿法替尼作为分子探针检测EGFR蛋白突变并不理想。

## 5 结语

细胞、动物实验及临床研究都已经证实了靶向EGFR蛋白分子探针在体成像的可能性，尤其是EGFR-TKI类分子探针。但由于对野生型EGFR或多或少具有结合能力，造成肿瘤组织与周围组织的靶/本比不够高，再就是亲脂性高，组织清除率快，不利于肿瘤组织内蓄积显像等缺点。而且所用标记核素如^11^C标记半衰期过短，不适合临床的广泛应用。因此出于临床转化应用的考虑，理论上较好的EGFR-TKI分子探针应该能用半衰期较长的核素标记（如^18^F），而且容易合成，同时兼具灵敏性高、特异性强、成像结果判定明确等特点。随着EGFR-TKI的不断研发，第三代产品如奥希替尼（AZD9291）作用机理明确，对*EGFR*突变蛋白的亲和力和特异性明显提高，对野生型EGFR作用极弱，可形成更高的靶/本比（40倍），故而值得探索将其标记成分子探针，研究其作为PET-CT示踪剂在体检测*EGFR*突变蛋白的可行性，以及通过其显像结果指导临床使用EGFR-TKI治疗NSCLC的可行性。

尽管理论上EGFR-TKI-PET不能区分*EGFR*突变的具体亚型，但应该能更好地预测EGFR-TKI的临床疗效。当然，从基础研究到临床使用还有很长的路，但是以靶向*EGFR*突变蛋白的分子探针为基础的分子影像为临床检测*EGFR*突变提供了一种全新的在体无创检测手段，为NSCLC的分子诊断提供了一个新的研究方向。
